# Targeting fin whale conservation in the North-Western Mediterranean Sea: insights on movements and behaviour from biologging and habitat modelling

**DOI:** 10.1098/rsos.231783

**Published:** 2024-03-06

**Authors:** Viola Panigada, Thomas W. Bodey, Ari Friedlaender, Jean-Noël Druon, Luis A. Huckstädt, Nino Pierantonio, Eduard Degollada, Beatriu Tort, Simone Panigada

**Affiliations:** ^1^ Tethys Research Institute, c/o Acquario Civico, Viale G.B. Gadio 2, 20121 Milano, Italy; ^2^ School of Biological Sciences, University of Aberdeen, Aberdeen AB24 3FX, UK; ^3^ Institute of Marine Sciences, Department of Ecology and Evolutionary Biology, University of California Santa Cruz, Santa Cruz, CA, USA; ^4^ Joint Research Centre, (JRC), European Commission, Ispra, Italy; ^5^ Centre for Ecology and Conservation, University of Exeter, Penryn, Cornwall, UK; ^6^ Associació EDMAKTUB, 08393 Barcelona, Catalonia, Spain

**Keywords:** satellite telemetry, cetacean, environmental predictors, foraging, Marine Protected Areas, Particularly Sensitive Sea Area

## Abstract

Biologging and habitat modelling are key tools supporting the development of conservation measures and mitigating the effects of anthropogenic pressures on marine species. Here, we analysed satellite telemetry data and foraging habitat preferences in relation to chlorophyll-a productivity fronts to understand the movements and behaviour of endangered Mediterranean fin whales (*Balaenoptera physalus)* during their spring–summer feeding aggregation in the North-Western Mediterranean Sea. Eleven individuals were equipped with Argos satellite transmitters across 3 years, with transmissions averaging 23.5 ± 11.3 days. Hidden Markov Models were used to identify foraging behaviour, revealing how individuals showed consistency in their use of seasonal core feeding grounds; this was supported by the distribution of potential foraging habitat. Importantly, tracked whales spent most of their time in areas with no explicit protected status within the study region. This highlights the need for enhanced time- and place-based conservation actions to mitigate the effects of anthropogenic impacts for this species, notably ship strike risk and noise disturbance in an area of exceptionally high maritime traffic levels. These findings strengthen the need to further assess critical habitats and Important Marine Mammal Areas that are crucial for focused conservation, management and mitigation efforts.

## 1. Introduction

Given the current rate of biodiversity loss [1], there is an urgent need to develop an understanding of the threats and conservation requirements of a wide range of species and ecosystems [2]. This challenge is particularly complex for wide-ranging, rare or cryptic species that are difficult to monitor, access or otherwise study [3,4]. The paucity of data on such species is often seen as one of the main barriers to developing effective evidence-based conservation and mitigation measures [5,6]. In this context, fast-paced technological advancements and analytical frameworks developed in recent decades are key tools for reducing these current knowledge gaps. The use of unmanned aerial vehicles (UAVs; [7,8]), environmental DNA (eDNA; [9]), remotely sensed imagery [10] and satellite-linked telemetry [11,12] has been critical in this regard, particularly within marine environments where regular observation would be otherwise logistically impractical.

Biologging, the practice of collecting and transmitting physical and biological information through dedicated sensors attached to animals [13], has been increasingly used in behavioural ecology in the context of animal movement, distribution and habitat use, providing insights into behavioural states [14] and home ranges of tracked individuals or wider populations. This knowledge is crucial to support the development of targeted spatially explicit management and conservation prioritization tools [11,15,16]. As the path to translate tracking data into policy is often slow and complex, researchers and policy makers within some regions—such as around the Mediterranean Sea—have yet to fully embrace this process [17]. Despite the considerable effort in the Basin to gain knowledge on the ecology of resident cetacean species and to promote their conservation [18], the use of such approaches remains limited [15,19–21].

**Figure 1.**
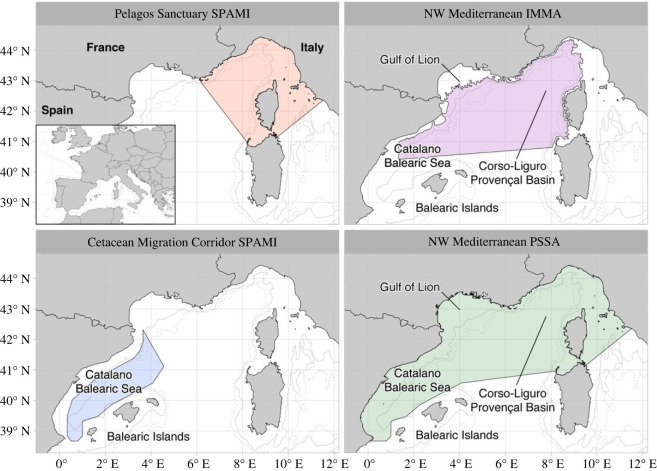
Extent and distribution of the main conservation regimes in the study area: the Pelagos Sanctuary for Mediterranean Marine Mammals (later also established as a Specially Protected Area of Mediterranean Importance; SPAMI); the North-Western Mediterranean Important Marine Mammal Area (IMMA); the Cetacean Migration Corridor SPAMI; and the North-Western Mediterranean Particularly Sensitive Sea Area (PSSA). The SPAMIs are MPAs with active management plans, while the PSSA only suggests voluntary measures. The IMMA, instead, carries no formal protection. The 1000 m and 2000 m depth contours are shown, respectively, with solid and dashed lines.

Fin whales are the only Mysticete to regularly occur in the Mediterranean Basin and constitute a genetically isolated sub-population from their North-East North-Atlantic (NENA) conspecifics [18,22], although exchanges through the Strait of Gibraltar have been increasingly reported [23,24]. The Mediterranean sub-population of fin whales is listed as Endangered in the latest International Union for the Conservation of Nature (IUCN) regional Red List assessment [25], in which a precautionary but continuing decline is inferred due to their exposure to anthropogenic activities. While impacted by a range of threats including epizootics [26], underwater noise [27] and climate change [28], there is general consensus that ship strikes are the leading cause of non-natural mortality [29–31], with the Mediterranean Sea ranked second globally for the number of ship strikes [31–33]. This region hosts some of the busiest traffic lanes in the world (approx. 30% of commercial shipping worldwide [25]), with the highest risk of collision posed by cargo vessels and, particularly, passenger ferries cruising at relatively high speeds (10–20 knots) [34]. To mitigate these threats, a suite of different management tools have been developed in the North-Western Mediterranean Sea (figure 1): the Pelagos Sanctuary for Mediterranean Marine Mammals Specially Protected Area of Mediterranean Importance (SPAMI) (hereafter ‘Pelagos Sanctuary'; [35]); the Cetacean Migration Corridor SPAMI; the North-Western Mediterranean Sea, Slope and Canyon System Important Marine Mammal Area (hereafter ‘NW Mediterranean IMMA'; [36,37]); and the North-Western Mediterranean Sea Particularly Sensitive Sea Area (hereafter ‘NW Mediterranean PSSA'), recently established by the International Maritime Organization (IMO; [38]).

While some movement data exist for Mediterranean fin whales [15,19,20], the understanding of this species' movement ecology remains limited. Evidence suggests that Mediterranean fin whales exhibit different movements compared to populations elsewhere, whereby they follow a contraction/dispersion pattern linked to seasonal variability in available foraging habitats within and outside of the Mediterranean Basin, rather than periodically moving between feeding and breeding grounds [18,21,39].

Fin whales congregate in the waters of the Corso-Liguro-Provençal basin and the Gulf of Lion [18,40] as the main summer feeding grounds and, in the last decade, they have also been consistently reported in the Catalano-Balearic Sea in spring [34]. However, it has never been assessed where or how these whales move after leaving this spring area.

In this study, we tracked fin whales with satellite-linked transmitters during the spring/summer months in the North-Western Mediterranean Sea, predicting that the individuals will move eastwards from the Catalano-Balearic Sea, following the seasonal evolution of summer foraging habitats in the Corso-Liguro-Provençal basin. To test our hypothesis, we examined (1) spring–summer movement patterns, (2) home ranges, habitat use and overlap with existing Marine Protected Areas (MPA) and other protection regimes. In addition to providing better understanding of fin whale movements and habitat use, our results will help identify important foraging hotspots which can help design monitoring actions and guide management [41].

## 2. Material and methods

### 2.1. Study area

In 2021 and 2022, data were collected in an area of about 2000 km^2^ in the Catalano-Balearic Sea between the North-Eastern Mediterranean coast of Spain (hereafter ‘Catalan coast’) and the northern Balearic Islands (figure 1). In 2023, fieldwork was carried out in the offshore waters of the Gulf of Lion. The Catalano-Balearic Sea is characterized by several submarine canyons, both in the shallow continental shelf (roughly 400 m deep) and in the deep pelagic waters (roughly 1500 m) [42]. The geomorphology of the area, in combination with the main current regimes, promotes primary production and results in high densities of Northern krill *Meganyctyphanes norvegica,* fin whales' main prey, along the shelf edge in spring [18,43]. This coincides with the regular observation of high numbers of fin whales along the Catalan coast in the spring months [34]. The Gulf of Lion presents similar oceanographic characteristics: the canyon-rich area allows for intense frontal and eddy activity at the continental shelf edge, when the water mixes with the open sea [34,43]. Moreover, the influx of nutrient-rich waters from the Rhone River makes the area highly productive with strong chlorophyll-a fronts, sustaining large aggregations of Northern krill [44,45].

### 2.2. Telemetry data collection and analysis

In 2021 and 2022, satellite transmitter deployments were carried out in late spring (May) to track the animals as they left their aggregation areas in the Catalano-Balearic Sea to move towards their summering grounds. Fieldwork in the Gulf of Lion in 2023 occurred at the beginning of the summer (June). Ten Argos Smart Position Only (SPOT5, SPOT6; Wildlife Computers Inc., Redmond, WA, USA) and one depth-recording (SPLASH/MK10) satellite tags in the Low Impact Minimally Percutaneous External-electronic Transmitters (LIMPET, moulds 260B and 260C; Wildlife Computers) configuration were deployed on the dorsal fins of whales using a 150 lb draw weight crossbow (Vixen Excalibur II). As in [46], the anchors were sterilized with isopropyl alcohol prior to deployment to minimize the risk of contamination and tissue infection. Following [15], only adult individuals—of no specific sex—were tagged after being visually assessed as healthy. To allow for an *a posteriori* qualitative evaluation of the position of the transmitter on the whale and the animal's immediate reaction to deployment, a GoPro Hero 8 camera was used, and a UAV (DJI Mavic Pro 2/Phantom 3 Pro) was flown above the RIB and the whale.

As attachment durations of LIMPET tags on fin whales are relatively short (ranging from a few days to six to eight weeks; A. Zerbini 2021, personal communication), the goal was to maximize the number of uplinks rather than to preserve battery life [47]. The instruments were therefore programmed to relay an unlimited number of transmissions per day over two daily temporal windows (hours: 6–11, 16–22 in 2021; 6–11, 17–23 in 2022; 4–11, 16–22 in 2023), totalling 11–13 h per day; no data were collected outside these intervals. Time windows were selected using the Argos Satellite Pass Analysis (Wildlife Computers) to predict satellite availability over the study area during May–July 2021, 2022 and 2023. The Argos system automatically applies the Kalman filtering location algorithm, and we did further manual filtering, omitting all poor-quality class Z and any terrestrial locations [47]. Moreover, due to the low relevance of the remaining portion of the track for the purpose of this study, the transmission of ID 232686 was cut-off in correspondence with the first day of distinct transit behaviour out of the study area (22 July 2023) and the subsequent points were omitted from the analyses.

Kalman-filtered data from the Wildlife Computers portal were additionally filtered and regularized with the package *aniMotum* [48] for the software for statistical computing R [49], which fits a continuous-time state-space model (SSM) and uses it to predict regularized positions along the most likely path taken by the whales, incorporating process and observation errors. The package internally re-projects lon lat data to a Universal Transverse Mercator (UTM) grid, which is provided as the default output [48]. To determine differences in movement behaviours, a correlated random walk was fitted to all tracked individuals' interpolated locations using a conservative maximum speed threshold of 7 m/s (based on [50–52]) to interpolate locations at regular intervals of 3 h. The time-step of 3 h was obtained through trial-and-error, by comparing Akaike Information Criterion (AICc), one-step ahead prediction residuals, and fits of different state-space models using time steps ranging from 1 to 24 h.

#### 2.2.1. Home range estimation

Home ranges, defined here as realized habitats, were assessed by calculating Kernel Utilization Distributions (UD)—the probability that an animal is found at a given point in space [53,54]—in each year, and in all three study years combined. Utilization Distributions provide the likelihood of an animal being present at a given point within its core (50% isopleths) and home (95% isopleths) ranges [54]; these usage hotspots can be critical to delineate areas of high conservation priority [16,55,56]. UDs were calculated through the *kernelUD* function in the *adehabitatHR* R package [57] in the WGS 84 Universal Transverse Mercator (UTM) 31 N and 32 N (EPSG:32631) projection using the regularized positions resulting from the SSM; a bivariate normal kernel was used, and the smoothing parameter was computed with the reference bandwidth, the *ad hoc* method [57]. UDs were calculated at a spatial resolution of 10X10 km to assess the minimum extent of the animals' distributional ranges and to measure the spatial intensity of use. Core and home range contours were computed by extracting the 50^th^ and 95^th^ percentile of each UD [58]. Since the calculation of UDs were based on regularized locations that are temporally equidistant, it intrinsically takes into account the time interval between relocations, thus, these can be considered the areas in which animals spent 50% and 95% of their time during the tracked period [59]. Furthermore, to evaluate spatial and spatial–temporal relations across the areas visited by the whales each season, and by each whale within each season, we calculated two indices of overlap: the per cent overlap index [60] among seasons/years to assess the spatial overlap of both core and home ranges in different years; and the Bhattacharyya's affinity index [61] among individual whales within each year. These indices respectively evaluate the proportion of animal *i*'s home range that is overlapped by animal *j*'s home range, and the affinity between two or more whales, i.e. whether they use space independently of one another or not. For the Bhattacharyya's affinity, the index values were derived based on the obtained UDs, considering a 95% level for home ranges and 50% level for core ranges. Therefore, 0.95 and 0.50 are the highest possible raw values of the index and, accordingly, values of 0.95 and 0.5 indicate the highest possible affinity for home and core areas, respectively. By using the core and home UDs of individual tracked whales, these indices were calculated using the function *kerneloverlaphr* in the *adehabitatHR* package [57].

#### 2.2.2. Behavioural analyses

To distinguish between two focal behaviours, the regularized positions from the SSM were used to fit a 2-state Hidden Markov Model (HMM) through a maximum-likelihood estimation analysis with the R package *momentuHMM* [62]. The two states are distinguished by degree of autocorrelation and turning angle; Area Restricted Search (ARS) is characterized by high turning angles and low autocorrelation in direction and speed [63,64]. Conversely, it is assumed that during transiting, turning angles should be closer to 0 and autocorrelation should be much higher [14]. In this context, we consider ARS a behaviour typical of species that feed on patchy resources, to maximize searching effort in the most profitable areas, with the identification of ARS locations revealing the presence of putative foraging areas [15,41,65].

Careful selection of starting values for the parameters for the step length and turning angle distributions is crucial in HMMs, to avoid convergence issues in the optimization of the likelihood. A gamma distribution was used to describe the step lengths (i.e. the Euclidean distance between two consecutive relocations; [66]), and a von Mises distribution described the turning angles (electronic supplementary material, figure S1) [67]. The Viterbi algorithm was used to estimate the most probable sequence of behavioural states [67].

#### 2.2.3. Environmental niche modelling

The overall tracking data were overlaid on the results of an ecological niche model (ENM) used to predict potential feeding habitats of fin whales in the Mediterranean Sea. The presented feeding habitat model in this paper is the third calibration after the first two in 2012 [39] and in 2017 [15], following the reprocessing of the chlorophyll-a archival data by NASA (R2022) and the inclusion of a substantially larger set of fin whale observations (5202 presence data). Unlike purely data-driven approaches that are strictly linked to the local environment surrounding each observation (e.g. statistical modelling approaches such as Generalized Additive Models or MaxEnt methods), the favourable feeding habitat is here centred on the occurrence of productivity fronts (high levels of horizontal gradient of chlorophyll-a); the habitat model is therefore deterministic and not stochastic. We used a cluster analysis (see details in the electronic supplementary material) to assess to which level of chlorophyll-a gradient, of chlorophyll-a and bathymetry, this species is particularly attracted (table 1). Productivity fronts are active for long enough (from a few weeks to a few months) to allow meso-zooplankton populations to develop [68] and have been shown to attract a wide variety of top predators [15,39,69–71]. Chlorophyll-a fronts (and associated high levels of chlorophyll-a gradients) are therefore considered to be explanatory variables (or proxies) for the distribution of fin whales when they are actively feeding [15] (see electronic supplementary material for a full description). Furthermore, the previous habitat analysis showed that this species in the western Mediterranean prefers avoiding relatively low and high chlorophyll-a levels and low bathymetry [15,39]. Chlorophyll-a levels and bathymetry were used here to exclude these unfavourable environments, centring the feeding habitat on specific levels of productivity fronts. While the shelf break is often marked by the presence of productivity fronts in that area (e.g. east–west part of the Liguro-Provençal current), productivity fronts may also occur elsewhere (e.g. west–east offshore part of the Liguro-Provençal current), we therefore privileged the direct detection of productivity fronts and have not selected the bathymetry gradient as an explanatory variable. To describe the characteristics of pelagic habitats, we collected and compiled geographical rasters containing the pixel values of these three environmental descriptors, as described in table 1.

**Table 1.** Environmental predictors of the fin whale feeding habitat model and original data resolution (model grid is 2.5-min = 1/24°).

environmental predictor	variable name	source and original resolution
water depth (m)	bathy	General Bathymetric Chart of the Oceans: http://www.gebco.net/ (1-min grid)
chlorophyll-a concentration (mg .m^−3^)	CHL	MODIS-Aqua sensor (R2022) https://oceandata.sci.gsfc.nasa.gov/ (2.5-min. grid, daily)
chlorophyll-a gradient (mg .m^−3^.km^−1^)	gradCHL	processed from CHL (see equation S1 in the electronic supplementary material) (2.5-min grid, daily)

Only the surface chlorophyll-a value (minimum and maximum favourable range), the horizontal gradient of chlorophyll-a (minimum value and linear fit to cumulative distribution of presence data) and water depth (minimum value in the inner shelf and reduced habitat suitability in the outer shelf and shelf break) were used in the fin whale foraging habitat. A thorough description of the fin whale feeding habitat that is associated with the ecological niche is provided in the electronic supplementary material. The daily habitat with a value from 0 to 1 is expressed in percentage of favourable occurrence (0 to 100%) once integrated in time, and above 30% defines hereafter the main habitat (or above 0.3 for the daily habitat).

## 3. Results

The tracked fin whales (*n* = 11) transmitted for a range of 8–48 days, with an average duration of 23.5 days and (s.d. = 11.3 days; table 2).

**Table 2.** Summary of satellite telemetry data from fin whales tracked in the North-Western Mediterranean Sea 2021 (*n* = 3), 2022 (*n* = 5) and 2023 (*n* = 3).

animal ID	decoded as	first uplink date	last uplink date	no. of raw locations	uplink duration (days)
212759	MK10	8 May 2021	22 May 2021	302	14
212761	SPOT6	6 May 2021	6 Jun 2021	473	31
212762	SPOT6	7 May 2021	31 May 2021	717	24
232681	SPOT6	12 May 2022	21 May 2022	209	9
232682	SPOT6	12 May 2022	07 Jun 2022	546	26
232683	SPOT6	14 May 2022	8 Jun 2022	449	25
232684	SPOT6	15 May 2022	7 Jun 2022	412	23
232685	SPOT6	15 May 2022	23 May 2022	109	8
232686^a^	SPOT6	4 Jun 2023	22 Jul 2023^a^	1078	48
232687	SPOT6	5 Jun 2023	21 Jun 2023	328	16
232688	SPOT6	5 Jun 2023	10 Jul 2023	764	35

^a^Transmission was cut-off and subsequent points were omitted from the analyses when the individual moved out of the study area.

**Figure 2.**
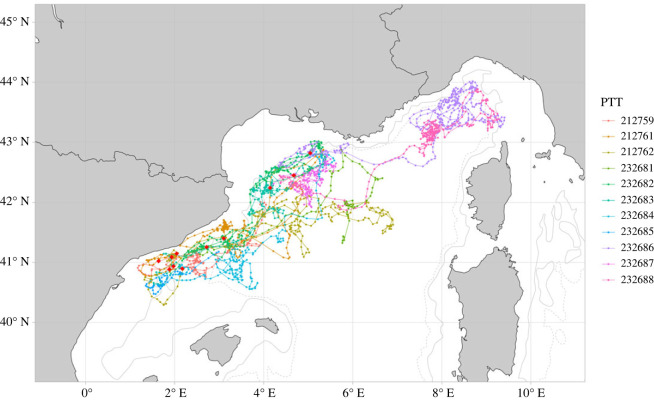
Filtered and regularized tracks of fin whales (*n* = 11) tagged in the North-Western Mediterranean Sea in May 2021 and 2022, and June 2023. First uplink locations are indicated with a red dot.

All individuals in all years showed consistent movements between the Catalano-Balearic Sea and the Corso-Liguro-Provençal Basin, with just one individual (ID: 232686) moving out of the study area, undertaking southward longer-range movements (figure 2).

### 3.1. Home range estimation

**Figure 3.**
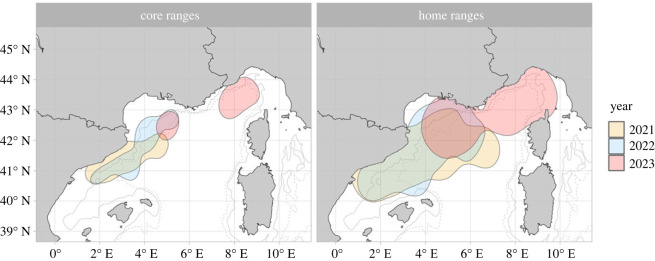
Core and home range areas—corresponding to, respectively, 50% and 95% isopleths—of Mediterranean fin whale satellite locations in 2021, 2022 and 2023. The 1000 m and 2000 m depth contours are shown, respectively, with solid and dashed lines.

The core and home ranges (electronic supplementary material, table S1, figure 3) showed limited variability of extent between 2021 and 2022, with a minimal (4%) decrease in area in 2022. There was a high degree of overlap between home ranges in 2021 and 2022 (per cent overlap index value = 0.8), and a more moderate overlap between core areas for the same years (index value = 0.5; figure 3; electronic supplementary material, figure S6). The same index shows that the 2023 core area, with tracking conducted over approximately one additional month, was substantially different to that of 2021 and 2022, with minimal overlap of respectively 2% and 18%. Similarly, the overlap of the 2023 home range area was also low, with an overlap of 25% with 2021 and 23% with 2022 (figure 3; electronic supplementary material, figure S6). When considering spatial–temporal relations, the Bhattacharyya's index for home ranges showed that, within each year, the tracked whales used a space with similar intensity. Bhattacharyya's index values for home ranges ranged between 0.1 and 0.9, with annual ranges of 0.4–0.9 in 2021, 0.1–0.9 in 2022 and 0.2–0.9 in 2023. When looking at core ranges, the Bhattacharyya's index showed a very limited overlap within animals tagged in the same year with values ranging between 0.0 and 0.5 (electronic supplementary material, figure S7). Only for those whales tagged in 2023, a more similar usage of space emerges with a Bhattacharyya's index value of 0.3. Overall, this suggests a relatively high independence for individuals within years.

### 3.2. Overlap with conservation areas

**Figure 4.**
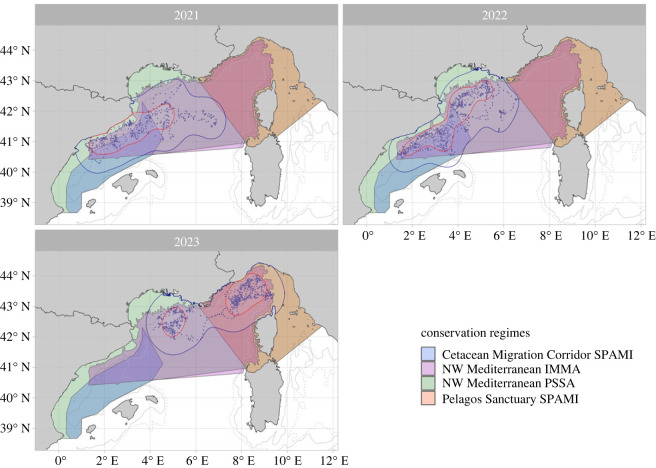
Spatial overlap of core (50% UD; in red) and home (95% UD; in blue) range contours of tracked fin whales overlapped with designated areas in the Mediterranean Sea: the actively managed Pelagos Sanctuary and Cetacean Migration Corridor, and areas with no formal protection, the NW Mediterranean IMMA and the NW Mediterranean PSSA. The 1000 m and 2000 m depth contours are shown, respectively, with solid and dashed lines. Points represent locations relayed from the satellite transmitters.

Overall, the movements of tagged whales across the three years fell within one or more areas with the potential to be managed for conservation (i.e. the NW Mediterranean IMMA and the NW Mediterranean PSSA; figure 4 and table 3); however, these areas currently carry no formal mandatory protection measures. Overlap with existing MPAs/SPAMIs (i.e. the Pelagos Sanctuary and the Cetacean Migration Corridor) with active management plans was limited (respectively, only two individuals tracked in 2023 entered the Pelagos Sanctuary, and less than 50% of the total home ranges for 2021 and 2022 overlap with the Cetacean Migration Corridor; figure 4 and table 3).

**Table 3.** Spatial overlap, expressed in per cent (%), between core and home ranges with existing spatially explicit protection tools in the North-Western Mediterranean Sea.

conservation regimes	year	core range (% overlap)	home range (% overlap)
Cetacean Migration Corridor SPAMI	2021	41.25	31.00
	2022	41.35	32.71
	2023	0	0
NW Mediterranean IMMA	2021	87.77	83.20
	2022	99.64	74.75
	2023	98.60	82.22
NW Mediterranean PSSA	2021	100	100
	2022	100	98.17
	2023	100	100
Pelagos Sanctuary SPAMI	2021	0	0.5
	2022	0	0
	2023	69.75	54.65

### 3.3. Behavioural analyses

**Figure 5.**
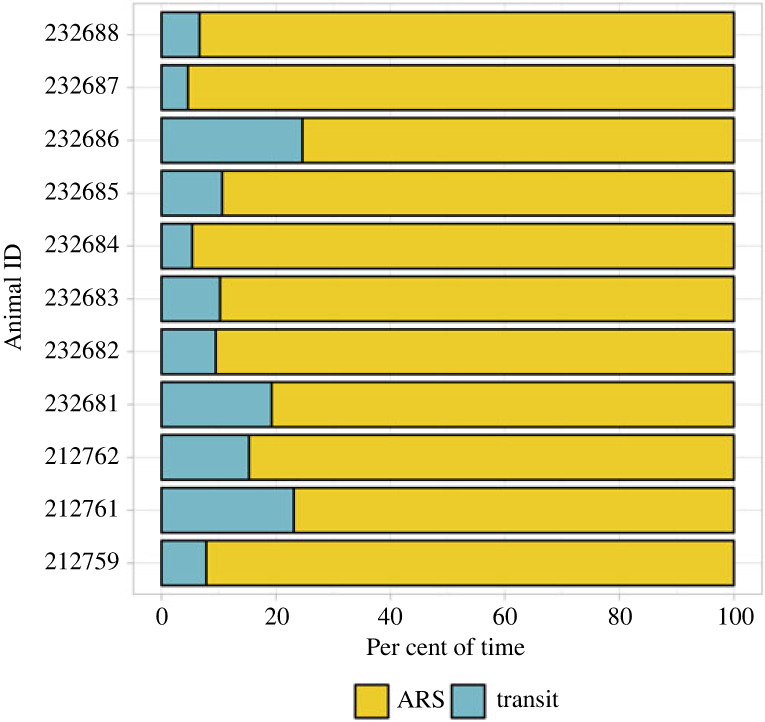
Activity budgets (i.e. percentage of time allocated to ARS or transit behaviours) of fin whales tracked in the North-Western Mediterranean Sea (*n* = 11).

The output of the HMM revealed that the individuals had consistent activity budgets, spending an average of 87.5% of their time engaging in ARS behaviour (median = 89.8%) (electronic supplementary material, table S2 and figure S8; figure 5). The spatial distribution of ARS locations was consistent across 2021 and 2022, with potential feeding hotspots off the coast of Catalunya and in the Gulf of Lion, while in 2023 whales mostly used the Gulf of Lion and the offshore waters of Western Liguria (electronic supplementary material, figure S8).

### 3.4. Insights from the prediction of feeding habitat

**Figure 6.**
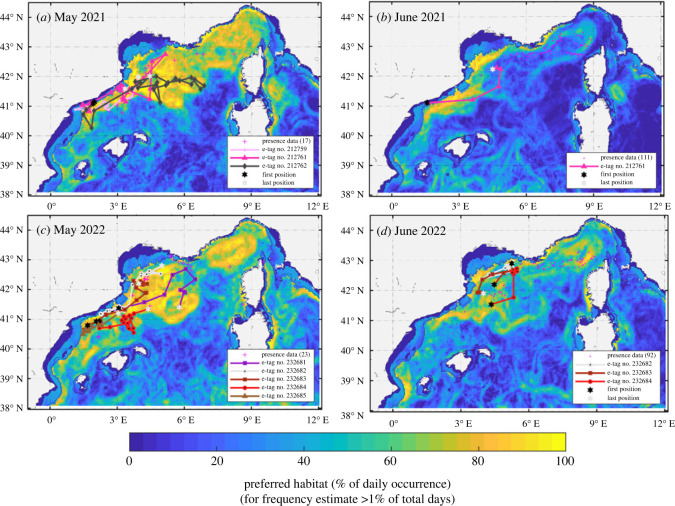
Eleven Argos satellite derived positions overlaid on monthly mean potential feeding habitat (frequency of occurrence for May–June 2021 [*a-b*] and May–June 2022 [*c-d*]). Note that only one high-quality tracking position per day is shown for clarity and pink dots indicate additional sightings. The potential habitat is derived from the daily detection of chlorophyll-a front, a range of surface chlorophyll-a content and a minimum water depth (see text and electronic supplementary material for details). The 200 m depth contour is shown.

The routes of fin whales tracked in May–June 2021–2022 were overlaid on the monthly mean potential feeding habitat to provide environmental context and improve the understanding of movements (figure 6). In all years, whales remained in the overall most productive areas, with individuals whose trajectories were heading towards less favourable feeding areas modifying their direction of movement to remain in areas with a higher frequency of chlorophyll-a fronts. The richest-potential feeding habitat was in the Corso-Liguro-Provençal current in May and June (figure 6) and, consistently across all 3 years, with higher habitat levels in the Catalano-Balearic Sea in June. The performance of the habitat model shows that it is relatively discriminant given the wide distribution of the species with, for example, 80% of observation data closer than 1 km to the main habitat (defined as greater than 30% favourable as a mean daily habitat over 3-days) (see electronic supplementary material, figure S4 for more information).

## 4. Discussion

By combining tracking data and the distribution of predicted foraging habitats, we were able to identify movement patterns and estimate habitat utilization of fin whales in the North-Western Mediterranean Sea. As predicted, limited interannual variation was observed in fin whale distribution for both tracking and potential habitat data, which was mirrored in the strong similarity between the 2021 and 2022 home ranges. However, tracked individuals in 2021–2022 spent less than 50% of their time in actively managed protected areas, instead occupying larger designated areas with no formal protection for 90–100% of the time (figure 4). In 2023, tracked whales spent 70% of their time in the managed protected area of the Pelagos Sanctuary SPAMI and occupied larger designated areas almost 100% of the time. While the relatively limited sample size and short tag deployment durations stress the need for further research, this study illustrates how the integration of multidisciplinary findings can serve as a baseline to enhance the informed management of this sub-population of fin whales [15,16,47].

### 4.1. Home range estimation and potential feeding habitats

The distribution of fin whale core ranges across years revealed recurring usage hotspots along the Catalan coast, providing further evidence of site fidelity in the Catalano-Balearic Sea [34] and the pelagic waters of the Gulf of Lion [15,39]. Large areas with high intensity of use were also highlighted in the Westernmost sector of the Pelagos Sanctuary in 2023, a well-known summer feeding area for the species [72]. While both core and home ranges show relatively little variation across years in their extent and location (figure 3 and electronic supplementary material, figure S6), for the summers of 2021 and 2022, we observed low within-year affinity among whales (i.e. high independence among animals in the use of space; electronic supplementary material, figure S7). Feeding is considered the primary factor driving the gathering of fin whales in the North-Western Mediterranean Sea [18]. In this context, the high within-year independence among whales might indicate that precise locations, specific nutrient requirements or ability to find prey could vary greatly from individual to individual. Equally, it could be the result of variation in principle foraging strategies or competitive abilities, resulting in individuals occupying patches of different productivity, as seen in many other marine organisms [73–77]. Such individual variability in traits including behaviour or physiology can result in biased estimates from relatively small sample sizes. Undoubtedly, increasing the annual research effort and the number of tag deployments per season would facilitate robust estimation of distribution patterns and the understanding of animals' behaviour in a specific area. The different season and areas where fieldwork took place (2021–2022 in the Catalano-Balearic Sea, 2023 in the Gulf of Lion) may account for the mirrored differences in habitat use and home ranges of the tracked individuals.

In general, both home and core ranges encompass critical habitats for fin whales in the North-Western Mediterranean Sea and agree well with prior knowledge on the presence and behaviour of fin whales in this portion of the Basin. Recent summer wide-scale aerial surveys [78–80], as well as studies based on photographic mark recapture [40,81] and visual [82] and acoustic surveys [27,83], show a consistent seasonal use of the Balearic Sea, the slope, and offshore waters of the Gulf of Lion and the Corso-Provençal-Ligurian Basin for feeding purposes. In this context, our study demonstrates that animal-borne tracking technology can be used to support and consolidate other approaches.

We also found that home ranges defined by the tracking data agreed strongly with independent habitat predictions based on the occurrence of productivity fronts. Accordingly, this provides robust elements for defining important habitat features that comprise core ranges in this area, season and species. Overall, only portions of the suitable feeding habitats and home ranges identified across the years in this study are currently protected as MPAs for cetaceans—the Cetacean Migration Corridor and the Pelagos Sanctuary SPAMIs. Instead, while much of the feeding and realized habitats fall within areas delineated for their importance for these species, such regions have no protection or mitigation measures in place, yet they have high volumes of vessel traffic and therefore have an increased risk of whale-vessel collisions [44]. Further research that expands the spatial–temporal scope of study is recommended to detail how these factors may change across and among years and to reduce potential biases [84].

### 4.2. Behavioural analyses

Results from the behavioural analyses suggested that, overall, the tracked fin whales predominantly engaged in ARS (mean: 87.5% of their time), only using transit behaviour to move between presumably profitable feeding areas. Owing to the patchiness of krill distribution, it is expected that once the rate of prey intake decreases in an area, a predator will move in a relatively straight path in search of the next prey patch [85]. This purposeful movement between patches is classified by the HMM as transit. Residency in foraging suggests the occurrence of multiple feeding events in the same patch. This was supported by visual observations of dense concentrations of krill species, with fin whales lunge feeding at the surface, characterized by tight turns and repeated strong vertical diving, and frequent defecation episodes [18,45,86] (V. Panigada 2022, personal observation). Using an HMM approach enables the identification of large-scale ARS zones that align with the productivity front-derived habitat in the study area (figure 6; electronic supplementary material, figures S8 and S9). The observed interannual variability of the productive front occurrence (electronic supplementary material, figure S9) likely contributes to slight differences in the distribution of individuals over the years in the same season and region.

### 4.3. Conservation actions: present and future

While about 6% of the Mediterranean Sea is technically protected, MPAs with full and high levels of protection, known to deliver ecological benefits, cover only 0.23% of the Basin [87]. Moreover, in 95% of the ‘technically' protected areas, there is no difference between the regulations inside the MPA and those outside of it [87]. This is in clear disagreement with the ‘theory of change' proposed by the ‘Kunming-Montreal Global Biodiversity Framework' (i.e. implementing urgent policy action at all levels to reverse trends that have exacerbated biodiversity loss by 2030; [88]). This Framework, together with the recent ‘30 × 30' initiative (CBD COP15; [88]), provides legally binding frameworks and new opportunities to protect cetaceans in the Mediterranean Sea. However, conservation benefits to wide-ranging cetacean species can be challenging due to the intrinsic difficulties of identifying and properly managing migration corridors, which may be in Areas Beyond National Jurisdiction (ABNJ) [89,90].

The fin whale is the cetacean species most often affected by ship strike in the Mediterranean Sea and globally [29,31,91]. Regular cargo and ferry routes run between Barcelona and the Balearic Islands, France, Tunisia and Italy, heavily intersecting the whales' core and home ranges identified in this study. Fatal collisions with fin whales have been reported to be more numerous in the warmest months in the Pelagos Sanctuary [29] and the Strait of Gibraltar [92], agreeing with the species' known summer feeding distribution [18,93] (figures 3 and 5).

The Cetacean Migration Corridor SPAMI was designed to protect a putative migratory passage for fin whales to their feeding grounds in the North-Western Mediterranean Sea. Our results suggest that the whales use it as a seasonal foraging, rather than a transiting, ground in its northern part (greater than 40.5 N; see also [94]). On top of the mismatch in expected and observed behaviour in the Migration Corridor, the benefits of this SPAMI to the fin whales that spend the spring months in the Catalano-Balearic Sea waters are limited: the animals' ranges overlapped with the SPAMI by less than 50% (figure 4), suggesting that, despite the relatively low sample size in this study (*n* = 11), the MPA might be poorly tailored to the movements and presence of this endangered sub-population in the spring-summer months.

The NW Mediterranean PSSA, which was recently established by the IMO, encompassed the entirety of the movements of the tracked fin whales until the end of transmissions. Our results show an excellent agreement between this PSSA and an area including most of the main foraging habitat predicted from a multi-annual mean (greater than 40% suitability for 2013–2022, electronic supplementary material, figure S9e). While recommended voluntary measures (e.g. speed reduction, increased observer surveys and reporting of cetacean sightings and collisions; enhanced awareness and specialized training of vessel crews) are already in place within the PSSA, more restrictive Associated Protective Measures (APM) should also be proposed under the framework of the IMO, prioritizing specific measures to avoid whale-vessels collisions [95]. The NW Mediterranean Sea, Slope and Canyon System IMMA also overlaps substantially with potential foraging habitat all-year-round, and especially when it is restricted in summer (electronic supplementary material, figure S9) [18]. In this context, our results, both in relation to tracking data and potential foraging habitat, validate the IMMA process towards informing the designation of the NW Mediterranean PSSA and potentially other statutory conservation and mitigation tools. For example, combining our spatial intensity of use and habitat modelling can inform the identification of critical areas for conservation through spatial prioritization [16]. Moreover, the Agreement on the Conservation of Cetaceans of the Black Sea, Mediterranean Sea and contiguous Atlantic area (ACCOBAMS) and the International Whaling Commission (IWC) have recently engaged in drafting a Conservation Management Plan (CMP) for Mediterranean fin whales. The overall goal of this CMP is to manage anthropogenic pressures to maintain a favourable conservation status throughout the species’ historical range. As the Mediterranean datasets become more robust, the data presented in this paper will be crucial when liaising with the relevant stakeholders to discuss the foreseen actions in the CMP and inform effective management efforts to increase the protection of fin whales in the entire Basin.

## 5. Conclusion

The integration of tracking data, behaviour, and modelling of potential foraging habitat provides valuable contributions towards the identification of high-risk areas for this species, where specific conservation/mitigation actions should be targeted. These findings, together with existing long-term survey data, reveal considerable foraging site fidelity to main feeding habitats, enabling the establishment of a tangible protection regime, with appropriate mitigation measures, within the boundaries of the recently established NW Mediterranean PSSA. Spatial management is recommended, for example, through dynamic seasonal regulations in the spring/summer months, when whale density is highest, because of main foraging habitat contraction. Despite the relatively small sample size, the limited temporal scale, and the intrinsic difficulties with large whale biologging, these data help identify specific areas which are critical for the species and where, until now, limited information existed (i.e. the Balearic Sea), and where management actions are urgently needed, especially in relation to vessel strikes (e.g. speed restrictions). This research finally reinforces the need to expand monitoring efforts of marine species by implementing a suite of multidisciplinary techniques at the Basin level, to integrate, *inter alia*, body condition and fine-scale behavioural assessments alongside conventional biologging. Such additional data will also contribute to the assessment of the potential for loss of habitat due to increasing maritime traffic noise and/or rising sea surface temperatures.

## Data Availability

Data available from the Dryad Digital Repository: https://doi.org/10.5061/dryad.h18931zsv [96]. Supplementary material is available online [97].
